# Enhancement and suppression of turbulence by energetic-particle-driven geodesic acoustic modes

**DOI:** 10.1038/s41598-017-17011-y

**Published:** 2017-12-01

**Authors:** M. Sasaki, K. Itoh, K. Hallatschek, N. Kasuya, M. Lesur, Y. Kosuga, S.-I. Itoh

**Affiliations:** 1 Research Institute for Applied Mechanics, Kyushu University, Kasuga, 816-8580 Japan; 2 Research Center for Plasma Turbulence, Kyushu University, Kasuga, 816-8580 Japan; 3 Institute of Science and Technology Research, Chubu University, Kasugai, 487-8501 Japan; 4 Max-Planck-Institute for Plasma Physics, 85748 Garching, Germany; 5 Lorraine University, Institut Jean Lamour, Nancy, 54-506 France

## Abstract

We propose a novel mechanism of enhancement of turbulence by energetic-particle-driven geodesic acoustic modes (EGAMs). The dynamics of drift-wave-type turbulence in the phase space is investigated by wave-kinetic equation. Spatially inhomogeneous turbulence in the presence of a transport barrier is considered. We discovered that trapping of turbulence clumps by the EGAMs is the key parameter that determines either suppress or enhance turbulence. In regions where turbulence is unstable, EGAM suppresses the turbulence. In contrast, in the stable region, EGAM traps clumps of turbulence and carries them across the transport barrier, so that the turbulence can be enhanced. The turbulence trapped by EGAMs can propagate independent of the gradients of density and temperature, which leads to non-Fickian transport. Hence, there appear a new global characteristic velocity, the phase velocity of GAMs, for turbulence dynamics, in addition to the local group velocity and that of the turbulence spreading. With these effect, EGAMs can deteriorate transport barriers and affect turbulence substantially. This manuscript provides a basis to consider whether a coherent wave breaks or strengthen transport barriers.

## Introduction

Problems including interactions between micro-turbulence and coherent waves are ubiquitous in a variety of systems^1,2^. The coexistence of global-coherent Alfven wave and micro-turbulence has been observed at the surface of the sun, which could link with the coronal heating problem^3^. Turbulence in planetary atmospheres generates zonal flows^2^, such as jet stream on the earth, stripes of Jupiter, super rotation on Venus, and tachocline on the sun^4^. In magnetically confined plasmas, geodesic acoustic modes (GAMs), which are oscillatory zonal flows, have attracted much attention as coherent waves, because GAMs are expected to suppress turbulence by their velocity shear^1^. Actually, the suppression of turbulence and transport have been reported in turbulence simulations^5^. Experimental study has shown that the transition to high confinement state is accompanied by GAMs^6^. However, recently, the enhancement of turbulence by GAMs has been observed in first principle simulations, with the subsequent destruction of a transport barrier^7–9^. In this way, GAMs can either mitigate or enhance the turbulence. This dual effect of the GAMs on turbulence requires theoretical investigation.

We investigate the phase-space dynamics of spatially inhomogeneous turbulence in the presence of GAMs. GAMs are driven not only by turbulence^5,10^ but also by energetic particles (EPs), which are called EGAM^11–14^. Turbulence driven GAMs suppress turbulence, which can be understood in terms of energy conservation (the total energy of GAMs and turbulence is conserved). In contrast, the impacts of EGAMs on turbulence is not clear, in particular because EGAMs obtain their energy from EPs, not from turbulence. Actually, EGAMs have been reported to enhance turbulence in spite of the fact that EGAMs have velocity shears^7,8^. In order to clarify the impact of finite-frequency zonal flows on turbulence, we focus on EGAMs. The phase-space dynamics results in trapping of turbulence wave-packets by EGAMs^15^. We found that the trapped turbulence wave-packets leak across the transport barrier. As a result, turbulence is enhanced by EGAMs in the stable region, while turbulence suppression is obtained in the unstable region. We discovered that trapping of turbulence clumps by the EGAMs is the key parameter that determines either suppress or enhance turbulence. The propagation of the turbulence is ballistic, with the phase velocity of the EGAM. Thus, the turbulence propagation is in some sense independent from the background profiles such as the gradients of density and temperature. Thus, turbulence carried by the EGAMs shows non-Fickian transport properties. The propagation of trapped turbulence is different from processes such as turbulence spreading^16–18^, avalanches^19–21^ and others^22,23^. Hence, there appear a new global characteristic velocity, the phase velocity of GAMs, for turbulence dynamics, in addition to the local group velocity and that of the turbulence spreading.

## Results

### Problem setting

**Figure 1:**
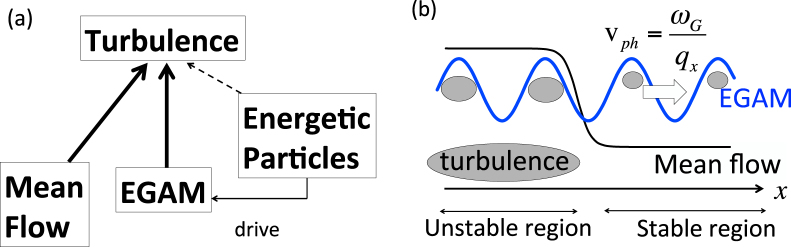
(**a**) Model on relationship between turbulence, mean flow, EGAM and EPs. (**b**) Model geometry of the inhomogeneous turbulence with the mean flow.

We consider the dynamics of spatially inhomogeneous turbulence with a transport barrier in the presence of EGAMs. We focus on the impact of EGAM on turbulence. As the first step, in order to simplify the problem, we neglect the direct effect of EPs on turbulence, as shown in Fig. 1(a). The radial profile of the flux surface averaged turbulence intensity is studied. The radial direction is set to *x*-direction. We consider a situation where the turbulence unstable region faces the stable region, and a transport barrier is localized at the boundary, as shown in Fig. 1(b). The transport barrier is simulated by a mean sheared flow. Turbulence is set to be linearly unstable (stable) inside (outside) the shear layer, respectively. The EGAM is assumed to have only a positive radial wavenumber, so the EGAM propagates from inward to outward the shear layer. It is noted that the EGAM has several branches; one has almost zero poloidal wavenumber *q*_*y*_ ≈ 0^11^ and another has a steep poloidal structure^24^. In this study, we focus on the branch with *q*_*y*_ ≈ 0, which is unaffected by the doppler-shift due to the mean sheared flow. This branch is driven by the resonance with the toroidally passing EPs, where we neglect the effect of the magnetically trapped EPs. In such a situation, the turbulence is governed by the wave-kinetic equation^25^,

1 $$\frac{\partial {N}_{k}}{\partial t}+\frac{\partial {\omega }_{k}}{\partial \vec{k}}\cdot \nabla {N}_{k}-\nabla {\omega }_{k}\cdot \frac{\partial {N}_{k}}{\partial \vec{k}}={\gamma }_{L}{N}_{k}-{\rm{\Delta }}\omega {N}_{k}^{2},$$

where *N*_*k*_ and *ω*_*k*_ are the normalized action and frequency of the turbulence, respectively^25^. The linear growth rate and the nonlinear decorrelation rate of turbulence are denoted by *γ*_*L*_, and Δ*ω*, respectively. Here time and space are normalized by ${\rho }_{s}^{-1}{V}_{d}$ and *ρ*_*s*_, where *V*_*d*_ is the diamagnetic drift velocity, and *ρ*_*s*_ is the ion gyro-radius measured by the sound velocity. It is noted that the typical frequency of the turbulence satisfies the relation, ${\omega }_{\ast }={\rho }_{s}^{-1}{V}_{d}\gg {\omega }_{G}\sim {\omega }_{EP}$, where *ω*_*EP*_ is the transit frequency of EPs. Thus, we assume that the resonance of the EPs with the turbulence is weaker than that with EGAMs. We treat *γ*_*L*_ as an independent parameter on EPs. We consider drift-wave-type turbulence, so *N*_*k*_ is given as ${N}_{k}={(1+{k}_{x}^{2}+{k}_{y}^{2})}^{2}|{\tilde{\varphi }}_{k}{|}^{2}$, and the frequency *ω*_*k*_ is

2 $${\omega }_{k}=\frac{{k}_{y}}{1+{k}_{x}^{2}+{k}_{y}^{2}}+{k}_{y}{V}_{y}(x,t),$$

where ${\tilde{\varphi }}_{k}$ is the normalized turbulent electrostatic potential, and *k*_*x*_, *k*_*y*_ are the turbulence wavenumbers. The turbulence frequency *ω*_*k*_ includes the doppler shift due to ${V}_{y}(x,t)$, which consists of the EGAM and the mean flow.

The turbulence trapping in the phase space was investigated in the previous studies^15,26^. Here, we compare the assumptions and results between these previous studies^15,26^ and the present work. In these studies, an analytic solution for a nonlinear wave pattern for zonal flows was obtained by considering the interaction between turbulence driven zonal flows and turbulence. The following assumptions were used; spatially homogeneous turbulence with a marginal stability condition was assumed (${\gamma }_{L}={\rm{\Delta }}\omega =0$), and the *k*_*x*_-spectrum distribution of *N*_*k*_ was prescribed. The dispersion relation for the drift wave in the paper^15^ is the same with that in this study, Eq. (2). In this study, prescribing the EGAM evolution, which is valid for the EP driven mode, we consider the spatially inhomogeneous turbulence by introducing the turbulence source (${\gamma }_{L},{\rm{\Delta }}\omega$), and the mean flow shear. We obtain the *k*_*x*_-spectrum evolutions of *N*_*k*_ without any assumption for the distribution. By considering the spatially inhomogeneous turbulence, we discover that the trapped turbulence clump can penetrate into the stable region with the ballistic propagation, which leads to enhancement of turbulence there.

### Trapping of turbulence by EGAM

**Figure 2:**
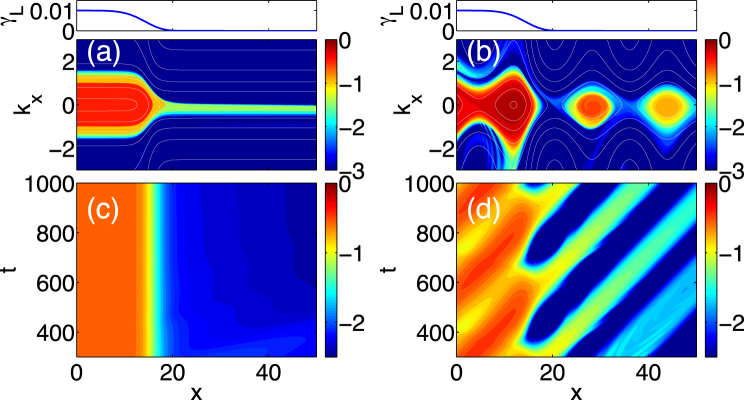
Snap-shots of *N*_*k*_ in the case of (**a**) ${V}_{G}=0$, and (**b**) ${V}_{G}=0.1$ at $t=800$ are shown. The time evolution of turbulence intensity is shown for the case of (**c**) ${V}_{G}=0$, and (**d**) ${V}_{G}=0.1$.

Let us describe the results obtained from the simulation. Figure 2 illustrates snapshots of *N*_*k*_ in the phase space $(x,{k}_{x})$, and the time evolution of the turbulence intensity $I(x)=\int {\mathrm{(1}+{k}_{\perp }^{2})}^{-2}{N}_{k}{d}^{2}k$ in the real space, where ${k}_{\perp }$ is defined as ${k}_{\perp }=\sqrt{{k}_{x}^{2}+{k}_{y}^{2}}$. The boundary between the turbulence unstable region and the stable region is *x* ≈ 20, and the shear layer is localized around the boundary. The results without the EGAM (*V*_*G*_ = 0) are shown in Fig. 2(a,c). Here, *V*_*G*_ is the amplitude of the *E* × *B* velocity of the EGAM. The *k*_*x*_-spectrum of the turbulence in the unstable region *x* < 20 has positive-negative symmetry with respect to *k*_*x*_. In the stable region, the *k*_*x*_-spectrum becomes asymmetric, and the turbulence exists only for *k*_*x*_ < 0, where the group velocity of the turbulence is positive. This outflow of the turbulence into the stable region can be understood by considering the motion of the quasi-particles (QPs). The equation of motion for the QPs is governed by Eq. (1) is^15,26^

3 $$\dot{x}(t)=\frac{\partial {\omega }_{k}}{\partial {k}_{x}}=-\frac{2{k}_{x}(t){k}_{y}}{{\mathrm{(1}+{k}_{x}{(t)}^{2}+{k}_{y}^{2})}^{2}},$$

4 $${\dot{k}}_{x}(t)=-\frac{\partial {\omega }_{k}}{\partial x}=-{k}_{y}\frac{\partial {V}_{y}(x,t)}{\partial x}{|}_{x=x(t)},$$

with ${k}_{y}=const$. Equations (3) and (4) correspond to the characteristics of Eq. (1), in the same way that, for example, the equations of motion of plasma particles correspond to the characteristics of the Vlasov equation. When the amplitude of the EGAM is zero and *V*_*y*_ is stationary, the motion of the QP becomes integrable. The drift frequency which includes the doppler shift due to the mean flow, Eq. (2), becomes a constant of motion. From this constant, the trajectories of the QPs are determined by

5 $${k}_{x}{(x)}^{2}=\frac{1}{C-{V}_{y}(x)}-\mathrm{(1}+{k}_{y}^{2}),$$

where *C* is a constant parameter, $C={k}_{y}^{-1}{\omega }_{k}$. Depending on the initial *k*_*x*_, the solution becomes bounded in the real space ($|{k}_{x}| < {k}_{c}=\sqrt{2{V}_{MF}(1+{k}_{y}^{2}){(1-2{V}_{MF}(1+{k}_{y}^{2}))}^{-1}}$), which corresponds to trapped motion in the unstable region, or becomes boundless ($|{k}_{x}| > {k}_{c}$), which corresponds to the transit motion across the shear layer. Here *V*_*MF*_ is the magnitude of the mean flow as defined in Eq. (12).

Next, we consider a case with finite amplitude EGAM. The numerical results with ${V}_{G}=0.1$ are shown in Fig. 2(b,d). The turbulence is modulated by the EGAM, temporally and spatially, in the unstable region, *x* < 20. In the stable region *x* > 20 (where there is no turbulence source, and the turbulence is affected by the nonlinear decorrelation), turbulence trapped by the EGAM forms islands in the phase space. As seen from the time evolution of the turbulence intensity in Fig. 2(d), the trapped turbulence propagates with the phase velocity of the EGAM, ${v}_{ph}={q}_{x}^{-1}{\omega }_{G}=0.05$. The dynamics of the turbulence can be understood by considering the motion of the QPs. When the time variation of the EGAM is negligibly small ${\omega }_{G}\ll {q}_{x}{v}_{g}$, *ω*_*k*_ becomes an adiabatic constant of motion. From Eq. (5), the trajectories of the QPs in the stable region can be categorized as

6 $$|{k}_{x}| < {k}_{sep}=\sqrt{\frac{{(1+{k}_{y}^{2})}^{2}{V}_{G}}{1-(1+{k}_{y}^{2}){V}_{G}}}\,:{\rm{trapped}},$$

7 $${k}_{sep} < |{k}_{x}| < {k}_{\ast }=\sqrt{\frac{1-(1+{k}_{y}^{2}){V}_{G}}{{V}_{G}}}:\,{\rm{transit}}\,.$$

When $|{k}_{x}| > {k}_{\ast }$, the QPs are reflected by the shear of the EGAM and the wavenumber diverges. The QPs with ${k}_{x} < {k}_{sep}$ are trapped by the EGAM, which results in an island.

The propagation distance of the turbulence in the stable region is derived. The trapped turbulence clump in the stable region at ${k}_{x}=0$ is governed by ${\partial }_{t}{N}_{k}=-{\rm{\Delta }}\omega {N}_{k}^{2}$. The solution of the envelope of the turbulence clump can be obtained as

8 $${N}_{k}=\frac{1}{{\omega }_{G}^{-1}{\rm{\Delta }}\omega {q}_{x}(x-{x}_{0})+1/{N}_{k\mathrm{,0}}}.$$

The trapped turbulence decays algebraically (unlike quantum tunneling) in space, with the typical scales, ${\omega }_{G}{\rm{\Delta }}{\omega }^{-1}{q}_{x}^{-1}$. The decay length derived here is consistent with the simulation results.

**Figure 3:**
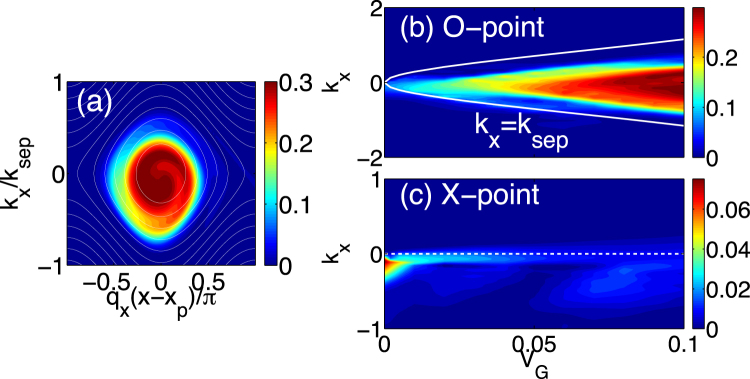
(**a**) Phase-space structure of the turbulence trapped by the EGAM in the stable region. The white lines are the contours of *ω*_*k*_. Dependence of *k*_*x*_-spectrums of the island at (**b**) O-point and (**c**) X-point on the amplitude of the EGAM are shown.

We describe two mechanisms by which the turbulence propagates in the stable region; one occurs without the EGAM (transit loss), and another is due to the turbulence trapping by the EGAM (trapped loss). We investigate conditions for trapped loss to be dominant. Figure 3 shows the change of the island shape as *V*_*G*_ varies. The island in the case of ${V}_{G}=0.1$ is shown in Fig. 3(a). Here the island is obtained from the ensemble average of islands, which satisfy ${q}_{x}{x}_{p}-{\omega }_{G}t=\mathrm{(1/2}+n)\pi$ (*n* is an integer, and ${x}_{p}=27$) with $t < 1000$. The amplitude dependence of the *k*_*x*_-spectra of ensemble averaged *N*_*k*_ at X-point, ${q}_{x}(x-{x}_{p})=\pi$, and at O-point, ${q}_{x}(x-{x}_{p})=0$, are shown in Fig. 3(b,c). With increasing *V*_*G*_, the intensity of the turbulence increases inside the island ${k}_{x} < {k}_{sep}$. When the EGAM amplitude is small, ${V}_{G} < 0.03$, the intensity inside the island is ambiguous, and the turbulence at X-point exists in ${k}_{x} < 0$. If the turbulence trapping is perfect, the turbulence does not exist at X-point. Thus, the transit loss is dominant when the amplitude is small. The conditions for the EGAM amplitude that the trapped loss becomes important can be understood as follows. Two conditions are necessary for the trapped turbulence to leak into the stable region. The first condition is that the turbulence is trapped by the EGAM. The trapped turbulence moves within the EGAM island with the bounce frequency ${\omega }_{b}=\sqrt{2{k}_{y}^{2}{q}_{x}^{2}{V}_{G}{\mathrm{(1}+{k}_{y}^{2})}^{-2}}$. In order to be trapped by the EGAM, the QPs should have a bounce period shorter than the EGAM period. Thus, the trapping condition is ${\omega }_{G} < {\omega }_{b}$. This condition can be written as

9 $${V}_{G} > \frac{{\omega }_{G}^{2}{(1+{k}_{y}^{2})}^{2}}{2{k}_{y}^{2}{q}_{x}^{2}}.$$

For our parameters, this condition is ${V}_{G} > 0.005$. When ${\omega }_{b}\sim {\omega }_{G}$, there is a possibility that the turbulence clump itself directly resonates with EPs. In such a situation, the trapping condition for the turbulence clump, Eq. (9), may be modified qualitatively. However, this effect is beyond the scope of this study, and we neglect it here. The second condition is that the trapped turbulence crosses the transport barrier. In order to leak across the shear layer, the bounce period should be shorter than the the transit time of the shear layer $\tau ={q}_{x}{\rm{\Delta }}{\omega }_{G}^{-1}$, where Δ is the width of the shear layer. Thus, the condition is

10 $${V}_{G} > {(\frac{\pi {\omega }_{G}}{{q}_{x}{\rm{\Delta }}})}^{2}\frac{{(1+{k}_{y}^{2})}^{2}}{2{k}_{y}^{2}{q}_{x}^{2}}.$$

When both conditions, Eqs (9) and (10), are satisfied, the trapped loss is important even when the curvature of the EGAM is smaller than that of the mean flow. Substituting the simulation parameters, the overall condition is ${V}_{G} > 0.02$, which is consistent with the simulation results. Actually, the curvature of the EGAM is always smaller than that of the mean flow in the present simulation, ${V}_{G} < {({q}_{x}{\rm{\Delta }})}^{-2}{V}_{MF}$. If ${V}_{G} < {({q}_{x}{\rm{\Delta }})}^{-2}{V}_{MF}$ is satisfied, the curvature of the mean flow is completely suppressed by the EGAM, depending on the phase, and even the turbulence which corresponds to the transit QPs can leak into the stable region with the EGAM frequency.

**Figure 4:**
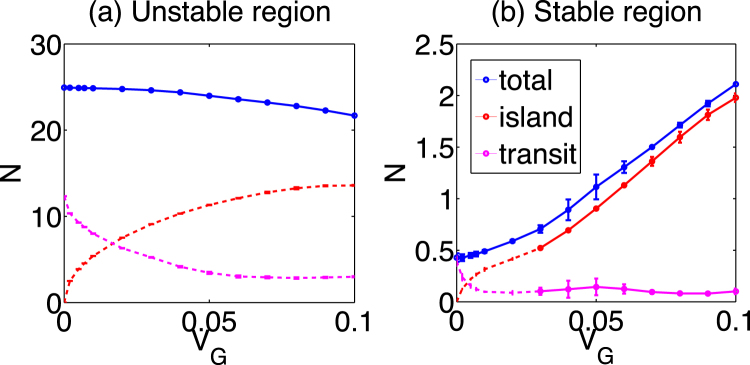
Amplitude dependence of the turbulence intensity at (**a**) *x* = 0 (unstable region) and (**b**) *x* = 27 (stable region). The blue, red and magenta lines indicate the total turbulence intensity, that inside the trapped region, and that outside the trapped region, respectively.

We study the dependence of the turbulence intensity $N={\int }_{x-\pi {q}_{x}^{-1}}^{x+\pi {q}_{x}^{-1}}dx\int d{k}_{x}{N}_{k}$ in the unstable and stable regions on the amplitude of the EGAM. Figure 4(a) illustrates the dependence of the turbulence intensity at *x* = 0 (the unstable region) on the EGAM amplitude. The intensity *N* is calculated from the ensemble average, and the error bar is estimated from the standard deviation. The intensity *N*, which is calculated from the whole region in *k*_*x*_-space, decreases with *V*_*G*_, whose amplitude dependence is fitted to ${V}_{G}^{2}$. The amplitude dependence in the stable region, *x* = 27, is shown in Fig. 4(b). The turbulence intensity increases with *V*_*G*_, which clearly shows that the EGAM can enhance turbulence. The total *N* in the stable region scales as $N=\alpha {V}_{G}^{1.5}+{N}_{transit}$, where the first term corresponds to the trapped loss, *α* is a constant parameter, $\alpha \approx 50$, and ${N}_{transit}$ is the component of the transit loss. Within the hypothesis that the trapped loss is perturbatively added to the transit loss, this parameter dependence can be understood: the intensity of the trapped turbulence is proportional to *V*_*G*_ and the spectral width increases with ${k}_{sep}\propto \sqrt{{V}_{G}}$. Their product yields the amplitude to the power 1.5.

The present mechanism of turbulence enhancement is discussed below in the contexts of experiments, and first-principle simulations. In the LHD, large amplitude EGAMs are observed with amplitude $e{\varphi }_{G}/T\approx 1$. Using the experimental parameters, the condition for the trapped turbulence, Eq. (9), is estimated as $e{\varphi }_{G}/T > 0.08$, so that the trapping effect of the EGAM is important. The amplitude for the trapping condition is not so large that the trapping effect could be important even for the turbulence driven GAM, whose observed amplitude is around $e{\varphi }_{G}/T\sim 0.1$ ^27^. In the case of the gyrokinetic simulations by Zarzoso and others^7,8^, the shear layer is much narrower than the EGAM wavelength, ${q}_{x}{\rm{\Delta }} < 1$, which is similar to that in this letter. Thus, the trapped turbulence could leak into the stable region. The amplitude of the EGAM is around ${V}_{G}\sim 0.1-1$, so the intensity of the leaked turbulence is expected to be comparable to that in the unstable region from the scaling obtained in this study. Therefore, the EGAM has a significant effect on the turbulence in the stable region, which leads to a substantial deterioration of the transport barrier as reported in the reference.

Finally, the propagation property of the trapped turbulence is compared with the other processes. The propagation characteristics are summarized in Table 1. The trapped turbulence propagates with the phase velocity of the EGAM (propagation speed is independent on the background profiles such as density and temperature gradients). The increase of the turbulence precedes the change of the background profiles. Thus, the turbulence in the stable region shows the non-Fickian transport properties. This is a new mechanism of the turbulence propagation, which is diferent from the ordinary turbulence spreading or avalanches^16,18,19^. The turbulence intensity is determined by that of the trapped turbulence *I*_*trap*_, while the theoretical expression of the turbulence intensity of the avalanche has not been not reported. The radial flux of the turbulence clump by the trapped turbulence by the EGAM is ${{\rm{\Gamma }}}_{EGAM}={q}_{x}^{-1}{\omega }_{G}{I}_{trap}({V}_{G})$, and that by the turbulence spreading is ${{\rm{\Gamma }}}_{spread}\sim {V}_{d}|\tilde{\varphi }{|}^{2}$. The intensity of the radial flux by the EGAM can dominate that by the turbulence spreading when *q*_*x*_ is small, and/or the amplitude is large. The propagation distance of the trapped turbulence is ${\omega }_{G}{\rm{\Delta }}{\omega }^{-1}{q}_{x}^{-1}$, in which the turbulence decays algebraically, while the distance is around $10{\rho }_{i}$ in the case of the turbulence spreading, where the turbulence decays exponentially. Thus, the range area of the influence of the trapped turbulence can be a plasma size.

**Table 1:** Comparison of turbulence propagation processes. Variables are shown in a dimensional form.

	propagation speed	turbulence intensity	propagation distance
avalanche^19,20^	~*V*_*d*_	$< |\tilde{\varphi }{|}^{2}$	~*a*
turbulence spreading^16^	~*V*_*d*_	|*ϕ*|^2^	~10*ρ*_*i*_
EGAM	${q}_{x}^{-1}{\omega }_{G}$	${I}_{trap}({V}_{G})$	${q}_{x}^{-1}{\rm{\Delta }}{\omega }^{-1}{\omega }_{G}$

## Discussion

In conclusion, two novel effects of the EGAM on turbulence are proposed by studying the phase-space dynamics of turbulence. Spatially inhomogeneous turbulence in the presence of transport barrier is considered. In the turbulence unstable region, the shear of the EGAM suppresses the turbulence. On the other hand, the turbulence trapped by EGAM leaks into the stable region across the transport barrier. Hence, turbulence is enhanced by the EGAM. The condition for the wave-trapping effect to be dominant is obtained, which is consistent with the simulations. The trapped turbulence by the EGAM can propagate independently from the gradients of density and temperature, which leads to the non-Fickian transport. Hence, there appear a new global characteristic velocity for turbulence dynamics, in addition to the local group velocity and that of the turbulence spreading. This manuscript provides a basis to consider whether a coherent wave breaks or strengthen transport barriers.

## Methods

### Simulation conditions

A monochromatic *k*_*y*_-spectrum is assumed for simplicity^26^, and the time evolution of *N*_*k*_ is calculated in the phase space of $(x,{k}_{x})$. We prescribe ${\gamma }_{L}(x,{k}_{x})$ and ${V}_{y}(x,t)$ as

11 $${\gamma }_{L}\,(x,{k}_{x})={\gamma }_{0}\exp (-|\frac{x}{{\rm{\Delta }}x}{|}^{{n}_{x}})\exp (-|\frac{{k}_{x}}{{\rm{\Delta }}k}{|}^{{n}_{k}}),$$

12 $${V}_{y}(t,x)={V}_{G}\,\sin ({q}_{x}x-{\omega }_{G}t)+{V}_{MF}\{\tanh \,[\frac{(x+{x}_{0})}{{\rm{\Delta }}}]-\,\tanh \,[\frac{(x-{x}_{0})}{{\rm{\Delta }}}]-1\}.$$

Here, *V*_*G*_ is a given parameter, since the EGAM survives regardless of the presence of turbulence. We implicitly assume that the scale length of the EP-density is much longer than the wavelength of the EGAM. The calculation region is $|x| < {x}_{max},|{k}_{x}| < {k}_{x,max}$. Neumann type boundary conditions are chosen: the derivatives of *N*_*k*_ with respect to *x* and *k*_*x*_ are set to be zero at the boundaries. The simulations are performed with the set of the parameters;${x}_{max}=\mathrm{60,}{k}_{x,max}=6.0,$ ${k}_{y}=1,{\gamma }_{0}=0.01,{\rm{\Delta }}\omega =0.01,{\rm{\Delta }}x={x}_{0}=15,$ ${\rm{\Delta }}k=1,{n}_{r}=5,{n}_{k}=2,$ ${q}_{x}=0.4,{\omega }_{G}=0.01,$ ${V}_{G}=0-0.1,{V}_{MF}=0.12,{\rm{\Delta }}=2$. The initial condition for ${N}_{k}$ is given as ${N}_{k}(x,{k}_{x},t=\mathrm{0)}={\gamma }_{L}(x,{k}_{x})/{\rm{\Delta }}\omega$. We calculate the time evolution of *N*_*k*_ numerically.
